# The development, implementation and evaluation of clinical pathways for chronic obstructive pulmonary disease (COPD) in Saskatchewan: protocol for an interrupted times series evaluation

**DOI:** 10.1186/s12913-017-2750-x

**Published:** 2017-11-28

**Authors:** Thomas Rotter, Christopher Plishka, Mohammed Rashaad Hansia, Donna Goodridge, Erika Penz, Leigh Kinsman, Adegboyega Lawal, Sheryl O’Quinn, Nancy Buchan, Patricia Comfort, Prakesh Patel, Sheila Anderson, Tanya Winkel, Rae Lynn Lang, Darcy D. Marciniuk

**Affiliations:** 1 College of Pharmacy and Nutrition, University of Saskatchewan E3315 Health Sciences Building, 104 Clinic Place, Saskatoon, SK S7N 5E5 Canada; 2 Healthcare Quality Programs, Queen’s University School of Nursing, Kingston, Canada; 3 Regina Qu’Appelle Health Region, Regina, Canada; 4 College of Medicine, University of Saskatchewan, Saskatoon, Canada; 5 University of Saskatchewan Respiratory Research Centre, Saskatoon, Canada; 6 University of Tasmania and Tasmanian Health Organisation (North), Launceston, TAS Australia

**Keywords:** Chronic obstructive pulmonary disease, COPD, Clinical pathways, Care pathways, Critical pathways, Integrated care pathways, Care maps

## Abstract

**Background:**

Chronic obstructive pulmonary disease (COPD) has substantial economic and human costs; it is expected to be the third leading cause of death worldwide by 2030. To minimize these costs high quality guidelines have been developed. However, guidelines alone rarely result in meaningful change. One method of integrating guidelines into practice is the use of clinical pathways (CPWs). CPWs bring available evidence to a range of healthcare professionals by detailing the essential steps in care and adapting guidelines to the local context.

**Methods/design:**

We are working with local stakeholders to develop CPWs for COPD with the aims of improving care while reducing utilization. The CPWs will employ several steps including: standardizing diagnostic training, unifying components of chronic disease care, coordinating education and reconditioning programs, and ensuring care uses best practices. Further, we have worked to identify evidence-informed implementation strategies which will be tailored to the local context.

We will conduct a three-year research project using an interrupted time series (ITS) design in the form of a multiple baseline approach with control groups. The CPW will be implemented in two health regions (experimental groups) and two health regions will act as controls (control groups). The experimental and control groups will each contain an urban and rural health region. Primary outcomes for the study will be quality of care operationalized using hospital readmission rates and emergency department (ED) presentation rates. Secondary outcomes will be healthcare utilization and guideline adherence, operationalized using hospital admission rates, hospital length of stay and general practitioner (GP) visits. Results will be analyzed using segmented regression analysis.

**Discussion:**

Funding has been procured from multiple stakeholders. The project has been deemed exempt from ethics review as it is a quality improvement project. Intervention implementation is expected to begin in summer of 2017.

This project is expected to improve quality of care and reduce healthcare utilization. In addition it will provide evidence on the effects of CPWs in both urban and rural settings. If the CPWs are found effective we will work with all stakeholders to implement similar CPWs in surrounding health regions.

**Trial registration:**

Clinicaltrials.gov (NCT03075709). Registered 8 March 2017.

## Background

Chronic obstructive pulmonary disease (COPD) is a respiratory syndrome characterized by progressive, partially reversible airway obstruction and lung hyperinflation [1]. This results in increasingly frequent and severe exacerbations [1]. COPD results in a substantial human and economic burden both within Canada and internationally. Although best practices have been developed to minimize this burden [1–4], implementation of these practices is generally fragmented [3]. In response to this fact, this protocol outlines the process of improving quality of care in two Saskatchewan health regions through the implementation of locally tailored clinical pathways (CPWs) and the subsequent evaluation of these CPWs.

### Prevalence

COPD is most often caused by smoking tobacco [5], but is also associated with air pollution [5] and occupational exposures to dusts and chemicals [5]. The disease is somewhat unique in the fact that it is generally underdiagnosed [6, 7]. This has led to difficulties in estimating prevalence at both the international and national level. Estimates for worldwide prevalence of COPD range from 4% to 20% [6]. Similar trends can be seen in Canada where approximately 4% of Canadians self-report as being diagnosed [7] but estimates based on airflow obstruction suggest a prevalence between 12% and 17% depending on diagnostic criteria [7]. The province of Saskatchewan has a prevalence similar to the national self-reported rate with an age standardized estimate of 4.3% [8].

### Disease burden

Although prevalence is difficult to determine it is clear that COPD is responsible for an enormous human burden, both internationally and within Canada. This is illustrated by estimates suggesting that internationally the disease will be the third leading cause of death by 2030 [6]. Within Canada COPD accounts for the highest rate of hospital admissions among major chronic illnesses [9]. This results in substantial financial costs, with direct costs estimated at CAD $2.02 billion in 2010 [10], a number expected to increase to CAD $4.6 billion by 2030 [10]. These estimates more than double when indirect costs are taken into account [10].

### Evidence based recommendations

In order to minimize the burden of COPD, high quality guidelines have been developed [1–4]. These guidelines generally specify disease identification through spirometry, management through a combination of smoking cessation, vaccination, pharmacologic therapy and physical activity, and proper management of COPD exacerbations (aeCOPD) with pharmacologic therapy [11]. When implemented, these steps have shown substantial improvements in patient quality of life, as well as a reduction in healthcare utilization. These benefits have been demonstrated in the Saskatoon Health Region (SHR) where the application of best practices through the LiveWell COPD program resulted in a 65% reduction in hospital admissions, a 61% reduction in inpatient days and a 44% reduction in emergency room visits [12].

Although these results are promising, evidence suggests that the creation of guidelines is inadequate [13–15] as passive dissemination alone rarely results in changes in practice [3, 16, 17]. Estimates across the healthcare environment suggest that 30–40% of patients do not receive treatments with proven effectiveness [18].

### Clinical pathways (CPWs)

One promising method of minimizing this gap is the implementation of clinical pathways (CPWs). CPWs, also known as integrated care pathways or clinical protocols, are tools used by health professionals to guide evidence-based practice and improve the interaction between health services. They bring the available evidence to a range of healthcare professionals by adapting guidelines to a local context and detailing the essential steps in the assessment and care of patients [19, 20].

Evidence exists to support the use of CPWs to change behaviour and improve quality of care [14, 15, 21–23]. A Cochrane systematic review on the use of CPWs in hospitals indicates that CPWs reduce in-hospital complications and improve documentation [24]. Recent studies regarding the use of CPWs for the management of COPD follow this positive trend, a fact evidenced by reported reductions in: hospitalization rates [25], readmission rates [25], length of stay [26], in-hospital complications [26], and increased preventative consultations [27]. However, studies generally fail to show a reduction in mortality [25, 26].

A review specific to the use of CPWs to improve in-hospital management of aeCOPD also found indications of positive results [28]. The review demonstrates evidence of improvements regarding: blood sampling, daily weight measurement, arterial blood gas measurement, referral to rehabilitation, feelings of anxiety, length of stay, readmission, and in-hospital mortality [28]. However, the authors suggest that results should be interpreted with caution as the review only included four studies. Further, these studies used a heterogeneous group of indicators and often failed to report statistics [28].

Although this limited group of studies shows some evidence regarding the assessment of CPWs for COPD, little has been done to determine the effects of CPWs within the province of Saskathchewan where CPWs have already been implemented for hip and knee surgeries [29], urogynecology assessments [30], treatment of prostate cancer [29] and treatment of stroke [30]. To date none of these pathways have been rigorously evaluated [29, 30].

## Methods/design

### Aims of the CPWs

In order to improve knowledge translation and address the limited evidence on the effectiveness of CPWs we will implement and evaluate two CPWs for the treatment of COPD within the province of Saskatchewan. The first CPW will be implemented in a predominently urban health region (Regina Qu’Appelle; RQHR) and the second will be implemented in a rural health region (to be chosen following implementation in RQHR). The development and implementation of the CPW will utilize the following steps:

- Identify and current best practices for the diagnosis and management of COPD;
- Use idenfied best practices in combination with local resources in the development of two CPWs;
- Tailor evidence-informed implementation strategies to the local context with the goal of encouraging uptake of the developed CPWs;
- Implement and pilot test the COPD pathway in two different Saskatchewan health regions; one urban (RQHR) and one in a rural (to be chosen at a later date);
- Evaluate the effectiveness of the CPWs based on chosen outcomes;
- Improve on areas of weakness identified through the evaluation

These steps are described in detail through the remainder of the documents. By applying this process we hope to achieve the following goals:

- Increase quality of care;
- Reduce healthcare utilization;
- Increase guideline adherence

To determine if these goals are met, the project will utilize an interrupted time series (ITS) design with control groups. We will compare the health regions which implement CPWs to both the control groups and to pre-inteventions measures.

### Foundational activities

To encourage the realization of these goals, the project is based on a number of foundational activities. The most substantial of these has been work to develop the LiveWell COPD program in Saskatoon. The program was initiated in 1999, but has benefitted from numerous updates. The current delivery format was introduced in 2005. The pathways used in the program employ a number of steps to improve quality of care. These include: standardizing diagnostic training through Lung Association of Saskatchewan’s RESPTREC spirometry training program [31], implementing and unifying common components of chronic disease care in the health region, coordinating the provision of education and reconditioning programs, and ensuring disease specific care utilizes and delivers evidence-informed practices. In addition, the LiveWell COPD program has worked to standardize documentation and improve in-hospital identification of COPD patients. The knowledge and expertise gained through the development and implementation of the LiveWell COPD program will guide our development and implementation processes.

Further, we have developed a strong working relationship with key stakeholders in the Regina Qu’Appelle Health Region (RQHR). We are currently engaged with these stakeholders to complete an updated systematic review regarding the use of CPWs for COPD. To date this has resulted in publication of a systematic review protocol [32], identification of relevant studies and extraction of data from these publications. This work has been used to identify best practices in developing and implementing a CPW for COPD. These best practices will be used in CPW development and implementation in both the urban and rural health regions.

Additionally, we have worked with this group to map the relevant services available within RQHR. This was done in order to 1) ensure that the CPW developed is well adapted to the local context and utilizes and coordinates currently available services; and 2) allows the research team to identify current gaps in the care of COPD patients and departures from best practices.

### Implementation strategy

Based on data extraction outlined in the systematic review protocol [32] we identified 12 evidence-informed implementation strategies [24, 33–36]. A cursory description of these activities is presented in Table 1.

**Table 1:** Overview of evidence-informed implementation strategies

Implementation strategy	Description
Development	
Clinician involvement	Utilization of individuals from all relevant professional groups [24, 33].
Evidence based interventions	Development which emphasises the importance of linking recommendations to the scientific research that supports them, identified through rigorous systematic identification and appraisal of all relevant research [24, 33].
Local consensus processes	Inclusion of participating providers in discussion to ensure that they agree that the chosen clinical problem is important and the approach to managing the problem is appropriate [34].
Analysis and Implementation Planning	
Implementation team	Utilization of a multidisciplinary change team. This team should include representation from three different leadership levels: Senior Leadership, Clinical/Technical Expertise, and Front-line Leadership [24, 34].
Identification of potential barriers to change	Strategies to improve professional practice taking into account prospectively identified barriers to change [24, 35, 36].
Identification of practice gaps	Collection and analysis of data related to the need for the innovation; this assessment is used for: the description of usual care and its distance from evidence based care, outcomes of usual care, opinions from stakeholders on the needs for an innovation, and/or special considerations for delivering the innovation in the local context [24, 36].
Education	
Local opinion leaders	The use of providers nominated by their colleagues as educationally influential [24, 33, 35].
Educational meetings	The participation of healthcare providers in conferences, lectures, workshops or traineeships [35].
Educational outreach	The use of a trained individual who meets with providers in their practice settings to give information with the intent of changing the providers’ practice [34, 35].
Printed educational materials	The distribution of published or printed recommendations for clinical care, including clinical practice guidelines, audio-visual materials and electronic publications [34, 35].
Systems	
Audit and feedback	Any summary of clinical performance of healthcare over a specified period, which is intended to change health professional behaviour. Indexed by objectively measured professional practice or healthcare outcomes [24, 35].
Reminders	Patient or encounter specific information, provided verbally, on paper or digitally. This information is intended to prompt a health professional to recall information [24, 34, 35].

As CPWs require that interventions and implementation are adapted to the local context, we will develop an implementation scheme based on these strategies for each health region. We are currently working to create an implementation team in RQHR that includes a physician lead and other local stakeholders to tailor implementation strategies. A similar process will be used when the rural health region is identified. Chosen implementation strategies will be described in future publications.

### Key stakeholders

This collaborative research project incorporates key stakeholders to ensure the best possible outcomes. Three organizations are integral to the success. The first is the continuous quality improvement teams from each health region. Within the province, these have been referred to as the Kaizen Promotion Office or Kaizen Operations Teams. These teams are an essential resource in the process of tailoring interventions to the local context. Second, the Saskatchewan Health Quality Council (HQC) is imperative, as representatives are able to amalgamate and produce all data necessary for evaluation. Finally, leaders in the LiveWELL COPD program in Saskatoon play a significant role, as these individuals possess the historical knowledge necessary to inform the development and continuous refinement of the CPW. Additional stakeholders crucial to the realization of the project goals are: members of the Saskatchewan Ministry of Health, Lung Association of Saskatchewan and Telehealth services from each of the regions as well as local clinicians such as: primary care providers, respirologists, pharmacists and nurses. To encourage input from all stakeholders we have developed a representative working group from RQHR. A list of current members of the working group and their expertise is presented in Table 2. A similar team will be assembled once a rural health region is chosen.

**Table 2:** Working Group Members

Name	Position
Sheila Anderson	Director, Primary Health Care, Kaizen Operations Team
Margaret Baker	Executive Director Primary Health Care, Ministry of Health
Bree Calland	Program Development Educator, Respiratory Services
Patricia Comfort	Primary Health Care Manager, Chronic Disease Prevention and Management
Lori Garchinski	Executive Director, Medicine
Dr Rashaad Hansia	Urban Primary Health Care Physician Dyad Leader
Shannon Jackson	Manager, Respiratory / Internal Medicine Unit, Regina General Hospital
Rae-Lynn Lang	Manager of Therapies, Acute Care
Taryn Lorenz	Director, Medicine
Sheryl O’Quinn	Manager, Respiratory Services
Dr Prakash Patel	Respirologist
Erin Roesch	Director, Primary Health Care Decision Support
Dr Shabaz Sheikh	Respirologist
Roberta Weist	Director, HealthLine Saskatchewan
Dr Fouche Williams	Rural Primary Health Care Physician Dyad Leader
Tanya Winkel	Pharmacist, Acute Care

### Design

We propose a quantitative health services research project using an interrupted time series (ITS) design in the form of a multiple baseline approach with control groups [37]. This methodology allows post-intervention trends to be compared with pre-intervention trends as well as the control group. We will compare the health regions which implement CPWs (intervention group) to both health regions which have not implemented the CPW (control group) and to pre-inteventions measures. Specificially, we will look at changes in:

- Quality of care, measured by:
  - ○ Hospital readmission rates;
  - ○ Unscheduled visits (emergency department);
- Healthcare service utilization, measured by:
  - ○ Admissions rates;
  - ○ Length of stay;
- Adherence, measured by:
  - ○ Scheduled visits (primary care providers, specialists)

### Primary outcome

The primary outcome for the study will be quality of care provided in participating health regions. This will be operationalized using hospital readmission rates and emergency department (ED) presentation rates.

### Secondary outcomes

The secondary outcomes of the study will be healthcare utilization and guideline adherence. Healthcare utilization will be operationalized using hospital admission rates and hospital length of stay. Guideline adherence will be operationalized as scheduled primary care provider and specialist visits.

### Analysis

Analysis will be conducted using segmented regression analysis. This form of regression is used to control for pre-existing trends by estimating changes in intercept and slope after the introduction of the intervention. Both an immediate impact (change in intercept) and gradual changes over time (change in slope) can be detected [38]. This design is less susceptible to the common threats to internal validity that occur in most observational study designs, including maturation and regression to the mean.

We will report the β coefficients which measure the following variables: model intercept prior to implementation (β_0_), model slope prior to implementation (β_1_), model intercept following implementation (β_2_) and model slope following implementation (β_3_). In this analysis a statistically significant β_2_ coefficient which is smaller than β_0_ suggests a decrease in the rate following the intervention. Similarly, a statistically significant negative β_3_ coefficient suggests a decrease in the rate of interest.

### Sample size considerations

We used two years of existing readmissions data to conduct power calculations for the ITS design. This was done using the simulation approach developed by Zhang et al. [39]. We estimated the Mean Squared Error (MSE), baseline intercept, and trend in the pre-intervention period using the pre-intervention data. Further we assumed either moderate or strong autocorrelation (AR(1) = 0.4 or 0.8); a change in intercept of 0, and two-sided α = 5%. We varied the number of post-intervention observation points from 6 to 30 and calculated the required change in slope that can be detected with at least 80% power. The analysis of the pre-intervention data showed strong secular trends in the primary outcome measure. We anticipate that for most outcomes, monthly observations will be used; in the case of small denominators for some outcomes, we may need to use quarterly or semi-annual intervals.

Based on these calculations we will use 36 pre- and post-intervention data points for most outcomes (with a minimum of 6 pre- and post-intervention data points for some secondary outcomes). As each data point for the primary outcome will contain one month’s data this equates to three years of pre-intervention and three years of post-intervention data.

### Settings

In order to evaluate the interventions, each health region will be matched with a control. For the intervention implemented in the Regina Qu’Appelle Health Region (RQHR), Saskatoon Health Region (SHR) will act as the control. Rural health regions will be chosen following implementation in RQHR and will occur following stakeholder consultation. As with the urban setting, the chosen rural health region will be compared to an appropriate control health region.

### Data sources

Data regarding hospital admissions rates, hospital readmission rate and length of stay will be retrieved through the Discharge Abstracts Database from the Canadian Institute of Health Information (CIHI) [40]. Data regarding scheduled GP visits will be retrieved through the Saskatchewan Health Quality Council Database. Data will be retrieved at the health region level and disaggregated to the hospital level.

## Discussion

Funding for the research has been awarded by the Lung Health Institute of Canada, the Saskatchewan Ministry of Health and through private industry (Novartis). The Research Ethics Board deemed to the project to be exempt from ethics review as the work conducted is a quality improvement project using automatically conducted and de-identified data. The study has been registered through clinicaltrials.gov (NCT03075709). Intervention implementation is expected to begin in RQHR in June 2017 with preliminary meetings with rural stakeholders following. Data collection will conclude 18 months after the start of implementation for each health region.

Successful steps to improve quality of life and reduce healthcare utilization have already been taken in some Saskatchewan health regions; however, these successes have not yet been replicated across the province. This has left substantial disparities in health outcomes for COPD patients among the province’s health regions. We aim to take the first steps to abate these differences by implementing a CPW in two Saskatchewan health regions.

In addition to increasing the quality of care received by COPD patients, this project will also provide evidence on the effects of CPWs in both urban and rural settings. This is crucial to decision making as CPWs have not yet been evaluated within Saskatchewan. Further, the intervention will provide a rigorous evaluation of the use of CPWs to improve care for COPD patients, adding to the limited evidence base currently available. If the pathways are found effective in both urban and rural settings, we will work with all stakeholders to implement similar CPWs across all areas of the province.

## Abbreviations

aeCOPD: Exacerbations of Chronic Obstructive Pulmonary Disease
COPD: Chronic Obstructive Pulmonary Disease
CPW: Clinical Pathway
ED: Emergency Department
GP: General Practicioner
HQC: Saskatchewan Health Quality Council
ITS: Interrupted Time Series
RQHR: Regina Qu’Appelle Health Region
SHR: Saskatoon Health Region
